# Supplementary material for: Efficacy of Health Promotion Interventions Aimed to Improve Health Gains in Middle-Aged Adults—A Systematic Review
Source: Geriatrics (Basel). 2023 Apr 30;8(3):50. doi: 10.3390/geriatrics8030050 (PMC10204560; doi:10.3390/geriatrics8030050)
Supplement: Supplementary file 1 [file geriatrics-08-00050-s001.zip › geriatrics-2293927-supplementary.pdf]

Table S1. Summary table of studies included in the systematic review of the literature.

Title/Origin/Authors/Year	Participants	Methodology	Purpose	Intervention	Results
**Title - A randomized controlled trial comparing community lifestyle interventions to improve adherence to diet and physical activity recommendations: the VitalUM study.**<br>**Origin - Netherlands**<br>**Authors -**<br>**Hilde Marijke van Keulen, Gerard van Breukelen, Hein de Vries, Johannes Brug, Ilse Mesters**<br>**Year - 2021**	1629)<br>Average age 57.15 years (SD=7.13)	RCT	To compare the efficacy of various sociocognitive lifestyle interventions to improve adherence to dietary and physical activity recommendations	Implementation of a 12-month lifestyle intervention aimed at adherence to healthy lifestyle recommendations (fruit and vegetable consumption and physical activity)<br>It consisted of the following:<br>- 4 personalized communication letters (TPC);<br>- 4 motivational interview sessions by telephone;<br>- Combined intervention (sending 2 personalized communication letters and conducting 2 motivational telephone interviews); and<br>- Distribution of a pedometer and homework (at week 29).	For physical activity, compared with the absence of an intervention, three interventions were associated with better adherence to guidelines.<br>Regarding adherence to physical activity guidelines, participants in the group that received computer-printed communication letters (TPC) exhibited the most adherence, followed by participants in the group that received motivational interviews (MIT), participants in the combined intervention group (letters and motivational interviewing), and finally the participants in the control group.<br>A pedometer had no influence on adherence to the physical activity recommendations.<br>Regarding fruit and vegetable consumption, comparisons between participants of the different groups showed that those who most adhered to fruit and vegetable consumption were participants in the group that received TPC, followed by participants in the group that received motivational interviewing (MIT), participants in the group that received the motivational interview combined with TPC (letters and motivational interview) and finally participants in the control group.

**Title-Effects of Individualized Aerobic Exercise Training on Physical Activity and Health-Related Physical Fitness among Middle-Aged and Older Adults with Multimorbidity: A Randomized Controlled Trial. Origin - Taiwan Authors - Yi-Pang Lo, Shang-Lin Chiang, Chia-Huei Lin, Hung-Chang Liu, Li-Chi Chiang Year - 2020**	43 () Mean age – 64.4 (SD=7.9) years	RCT	To examine whether a 12-week individualized aerobic exercise training in a rehabilitation center combined with motivational telephone interviewing is effective in promoting physical activity and health-related physical fitness among middle-aged and older adults with multimorbidity	(1) The intervention group participated in a multidisciplinary individualized aerobic exercise training program for 12 weeks, lasting 30 to 50 minutes per session, combined with motivational telephone interviews, once a week, lasting 15 to 30 minutes. (2) The comparison group participated in motivational telephone interviews for 12 weeks, once a week, with duration of 15 to 30 minutes (3) The control group received the usual care and general physical activity recommendations for 12 weeks.	The results indicated that 12 weeks of individualized aerobic exercise training, combined with motivational telephone interviewing had significant beneficial effects on total physical activity, vigorous-intensity physical activity, dominant and nondominant hand muscle strength, and various cardiorespiratory fitness outcomes. Compared with participants in the control group, middle-aged and older adults with multimorbidity in the intervention group exhibited increased total physical activity and vigorous-intensity physical activity as well as increased walking and decreased sedentary time at 12 weeks.
**Title - Grenada Heart Project–Community Health Action to EncouraGe healthy Behaviors (GHPCHANGE): A randomized peer control group–based lifestyle intervention**. **Origin - Grenada Island Authors - Jacqueline Latina, Rodrigo Fernandez-Jimenez, Sameer Bansilal, Samantha Sartori, Rajesh Vedanthan,**	402 () Mean age 51.4 (SD= 14.5) years	RCT	To evaluate the efficacy of a 1-year peer group intervention as a cardiovascular risk reduction strategy using a composite score to measure cardiovascular health without laboratory or	The intervention consisted of health education conducted by peers. The "peer leader" was a layperson in the community, selected among motivated individuals willing to receive additional training to moderate the peer groups and participate in group meetings. The leaders underwent an additional three-hour leadership and communication	Regarding the primary outcome, in the postintervention period, the mean Fuster-BEWAT score was higher in the intervention group than in the control group. However, the difference between groups (intervention vs. control) in FBS change after one year of intervention was only 0.31 points.

**Marcelle Lewis, Claire Kofler, Marilyn Hunn, Francis Martin, Emilia Bagiella, Michael Farkouh, Valentin Fuster**<br><br>**Year- 2020**		imaging instruments	skills training session, in addition to guidance on the promotion of healthy behaviors. Peer leaders provided topics for discussion at the monthly meetings, for example,<br>low-salt diet and high blood pressure, diabetes prevention, coping with diabetes, strategies for stress, and smoking cessation. The leaders could adapt the themes to the interest of each group. Leaders were encouraged to promote 150 minutes of physical activity per week, the consumption of at least five fruits and vegetables daily, smoking cessation, and blood pressure management. The project manager made routine visits to each group to encourage and monitor attendance and receive feedback from the group leader. The control group received a series of educational interventions, namely, lectures, at the time of enrollment in the study, followed by self-management for 12 months. At the time of registration, participants were instructed on

				how to measure their weight and blood pressure accurately.<br>Blood pressure devices were distributed in the community for self-control (in health centers and in each parish).	
**Title - Health-related quality of life and physical activity level after a behavior change program at Norwegian healthy life centers: a 15-month follow-up.**<br>**Origin - Norway**<br>**Authors- Ellen Eimhjellen Blom, Eivind Aadl, Guri Kaurstad Skrove, Ane Kristiansen Solbraa, Line Merethe Oldervoll**<br><br>**Year - 2020**	524<br>Mean age – 52.7 (SD= 13.8) years	Prospective observational study (case series)	To investigate changes in quality of life related to health and physical activity after a 3-month intervention<br>To study the associations between changes in physical activity and health-related quality of life	Design of a personalized plan for behavior change that consisted of the following:<br>-Interventions based on salutogenic approaches complemented with motivational interviewing;<br>-Group healthy eating courses (five times, two hours);<br>-Group smoking cessation courses (six to ten meetings);<br>-Meetings covering topics such as clothing during exercise, motivation, and supervised exercise (cardiorespiratory training, strength training, and guided physical activity) at least twice a week.	All health-related quality of life dimensions improved significantly from baseline at the 3-month follow-up, and the improvements were maintained at 15 months for all dimensions except mental health, which showed further improvement from 3 to 15 months of follow-up.<br>The changes observed at 15 months were greater than 5 points for all dimensions except for body functioning and body pain.<br>Physical activity increased from baseline to 3 months but decreased from 3 to 15 months.<br>There were positive associations between changes in physical activity and quality of life, indicating that participants who increased their level of physical activity were more likely to improve their quality of life.
**Title - Randomized Controlled Trial of the SystemCHANGE Intervention on Behaviors Related to Cardiovascular Risk in HIV+ Adults**.	101 y<br>Mean age – 53 years intervention group 52.3	RCT	To test the effect of an intervention on lifestyle behavior (SystemCHANGE) , physical activity and diet quality	The intervention had two components: behavior change techniques and education on a healthy lifestyle.<br>It consisted of six standardized group sessions focused on	The intervention did not significantly improve the behavioral parameters of the primary lifestyle and did not increase physical activity; however, the participants in the intervention consumed less carbohydrates per day (mainly sugar-

**Origin - United States of America**<br>**Authors - Allison R. Webel, Shirley M. Moore, Chris T. Longenecker, Jackson Currie, Christine Horvat Davey, Joseph Perazzo, Abdus Sattar, Richard A Josephson.**<br><br>**Year - 2019**	(SD=7.39) years control group 53.3 (SD=7.36) years		among sedentary people with HIV	improving lifestyle behaviors (general advice on diet and exercise guidelines).<br>Improvements in usual care were also promoted through the distribution of information on healthy eating and physical activity as well as social telephone calls. All participants used an ActiGraph accelerometer and performed a complete 24-hour dietary collection at baseline and at 3 and 6 months.	sweetened beverages) and lost 0.732 kg of body weight, compared with 0.153 kg in the control group.<br>The primary activity outcome was time spent in moderate to vigorous physical activity, and the primary dietary outcome was the Healthy Eating Index.
**Title - Smart wearable devices as an intervention for healthy lifestyle and quality of life: a randomized psychological controlled trial**<br>**Origin - Taiwan**<br>**Authors - Hsin-Yen Yen**<br><br>**Year - 2020**	73 (age between 20-65 years)	RCT	To explore the effects of wearable smart devices on health-promoting lifestyles and quality of life	Intervention implemented over 3 months that consisted of the use of two different commercial smart devices (smart watches in an experimental group and smart bracelets in another experimental group), with different levels of complicated functions as a psychological intervention; the control group used a smartphone application.	In the experimental group, there was a more significant effect of exercise and interactions on self-actualization and stress management with the use of a smartwatch (smart watch) than with the use of a mobile application only.<br>The significant group-to-group interaction effect for self-actualization was also observed in the experimental group in which the participants used a smart bracelet.
**Title - Effect of a short-term lifestyle modification program on quality of life, anthropometric characteristics and CD4+ T cell count of HIV infected Patients in Tehran/Iran: A Randomized Controlled Trial**	27 (age between 20-58 years)<br><br>Average 37 years	RCT	To investigate the effects of a lifestyle modification program (PML) on quality of life, anthropometric	The participants were encouraged and taught to become more physically and mentally active, to choose healthier foods, to increase fruit and vegetables and to adopt	Participants who participated in the lifestyle modification program had significantly higher CD4+ T-cell counts than did the participants in the control group.<br>In the control group, the percentage of fat mass increased significantly from baseline

**Origin - Iran**<br>**Authors - Morteza Ghayomzadeh, Maede Sadat Etesami, Conrad P. Earnest, Sajjad Rezaei, James Wilfred Navalta, Leila Taj, Seyed Ahmad Seyed Alinaghi, M. Phil, Minoo Mohraz, Reza Gharakhanlou, Fabricio Azevedo Voltarelli**<br><br>**Year - 2019**		characteristics and CD4+ T-cell count in<br>People living with HIV (PLWH)<br>To provide education for and improve nutrition without imposing additional costs (such as the purchase of certain foods)	healthier habits, such as reducing the amount of time watching TV and increasing mental and physical activities during their free time instead of engaging in sedentary behaviors.<br>The participants engaged twice a week,<br>for 75 minutes, in recreational sports activities based on fun, such as table tennis and badminton, as well as mental and cognitive activities such as throwing darts, playing chess and<br>backgammon. After 75 minutes of physical exercise, participants had 30 minutes of health education class taught by a physical exercise physiologist.<br>The focus of these group education sessions was to increase daily physical activity and modify daily habits.<br>These sessions also covered healthier eating tips derived from the recommendation and positions of several international nutrition societies. These sessions included topics covering advice on hydration, eating more fruits	throughout the study; in the intervention group, there were no significant changes.<br>The percentage of lean body mass decreased in the control group; in the intervention group, it did not change.<br>In both groups, there was no difference in body weight and BMI between baseline and the end of the intervention, in none of the groups.<br>Participants in the intervention group achieved improvements in all SF-36 subscales in the total quality of life score at the end of 8-week intervention and study period; these scores did not improve for the participants in the control group.

				and vegetables, eating regularly, turning off electronic devices at meals, limiting the amount of refined and simple carbohydrates, and reducing fried foods and fatty foods and replacing them with whole grains and complex carbohydrates.	
**Title -Effectiveness of an Energy Management Training Course on Employee Well-Being: A Randomized Controlled Trial Origin - USA (Boston) Authors - Sai Krupa Das, Shawn T. Mason, Taylor A. Vail, Gail V. Rogers, Kara A. Livingston, Jillian G. Whelan, Meghan K. Chin, Caroline M. Blanchard, Jennifer L. Turgiss, Susan B. Roberts. Year - 2019**	220 (mean age 46 years)	RCT	To evaluate the effectiveness of an intervention designed to increase vitality and purpose in life, assessing changes in quality of life and health-related behaviors	The intervention (health and well-being program) consisted of an intensive well-being promotion program (in group) lasting 2.5 days. There was periodic follow-up at 6, 12 and 18 months. The intervention was performed by trained coaches who developed it in person, using a multidisciplinary approach rooted in performance psychology, exercise physiology and nutrition to help maximize energy and promote behavior change throughout life. The intervention mixed cognitive behavioral therapy and acceptance and commitment therapy to directly target the participants' thoughts, actions,	Compared with the control Group, the intervention group had a significantly greater 6-month mean change in the SF-36 vitality scale and scored in the highest categories for 5 of the 7 remaining SF-36 domains: general health, mental health, and absence of role limitations due to physical and social functioning problems. The intervention group also had greater improvements with respect to purpose in life and quality of sleep. No statistically significant changes were observed regarding weight, diet, physical activity or cardiometabolic risk factors.

				emotional processing and social interactions.	
**Title - One-year intensive lifestyle intervention and improvements in health-related quality of life and mental health in persons with type 2 diabetes: a secondary analysis of the U-TURN randomized controlled trial**.<br>**Origin - Denmark**<br>**Authors - Christopher Scott MacDonald, Sabrina M Nielsen, Jakob Bjørner, Mette Y Johansen, Robin Christensen, Allan Vaag, Daniel E Lieberman, Bente Klarlund Pedersen, Henning Langberg, Mathias Ried-Larsen, Julie Midtgaard**<br><br>**Year - 2021**	98 (mean age 54.6 years)	RCT	To investigate whether a 1-year intensive lifestyle intervention is superior in improving quality of life compared with standard care for people with type 2 diabetes (T2DM)	Before the baseline evaluations, the participants had their glucose, lipid and blood pressure values monitored by an endocrinologist. Both the intervention and control groups received standard care, which consisted of medical advice, lifestyle advice, and education about type 2 diabetes, provided by a nurse, at baseline and every three months during the 12 months of intervention.<br>In addition, the intervention group received two primary interventions for increasing levels of structured exercise (240-300 minutes/week and 2-3 resistance training sessions/week);<br>and individualized meal plans.<br>All training was performed in a group and supervised throughout the study.<br>Supervision was gradually reduced.<br>A clinical nutritionist prepared the individual meal plans, and the implementation was	The main outcome was the change in the Physical Component Score (PCS) from baseline to the 12-month follow-up.<br>Secondary outcomes included a change in the Mental Component Score (MCS) from baseline to 12 months (significant improvement from baseline to follow-up at 12 months).<br>The exploratory results includes a change in the SF-36 subscales, in the total MHC-SF score, in the MHC-SF categorical score, and in the WEMWBS, and change from baseline for the positive and negative affectation scores derived from the MSG at the 12-month follow-up.

				continuously discussed during group sessions (the same groups as the training). In addition, four support interventions were applied: increasing sleep duration, increasing levels of daily physical activity, self-control of behaviors related to the other interventions, and diabetes management and education.	
**Title -Randomized Trial of Exercise on Quality of Life in Women With Ovarian Cancer: Women's Activity and Lifestyle Study in Connecticut (WALC)** **Origin - USA** **Authors-Yang Zhou, Brenda Cartmel, Linda Gottlieb, Elizabeth A. Ercolano, Fangyong Li, Maura Harrigan, Ruth McCorkle, Jennifer A. Ligibel, Vivian E. von Gruenigen, Radhika Gogoi, Peter E. Schwartz, Harvey A. Risch, Melinda L. Irwin.** **Year - 2017**	113 (mean age: 57.3 years)	RCT	To examine the effect of a six-month intervention of aerobic exercise versus attentional control on HRQoL and ICR in women diagnosed with ovarian cancer To test the hypothesis that women randomly assigned to the exercise group experience improvements in HRQoL and FRC	The intervention involved a six-month program of moderate-intensity aerobic exercise, performed at home, facilitated by weekly telephone calls, to each participant, by a certified trainer. The intervention consisted of 150 minutes per week of moderate-intensity aerobic exercise, mainly brisk walking.	In both groups, in women who participated in physical exercise for 150 minutes or more and answered telephone calls, there was a change in quality of life in physical health. There was an improvement in cancer-related fatigue in women who exercised 150 minutes or more per week compared to those who exercised less. Women randomly assigned to the exercise group who answered 80% or more of the telephone calls had a statistically significant improvement in FACT-F, compared with women randomly assigned to the attentional control group who also answered 80% or more of the telephone calls. Relapse of the disease during the study had no significant impact on adherence to telephone calls or exercise.

	compared to women randomly assigned to the attentional control group	Time of diagnosis modified the effect of exercise versus control on PCS in women diagnosed for more than 18 months but not in women diagnosed for less than 18 months.<br>The effects of exercise on PCS and SF-36 Physical Functioning (SF-PF) varied from the PCS and SF-PF baseline levels. Those with worse baseline scores benefited more from exercise than did those with better baseline scores.
